# Factors Affecting Ultra‐Processed Food Consumption: Hedonic Hunger, Food Addiction, and Mood

**DOI:** 10.1002/fsn3.70248

**Published:** 2025-05-07

**Authors:** Özge Mengi Çelik, Ümmügülsüm Güler, Emine Merve Ekici

**Affiliations:** ^1^ Department of Nutrition and Dietetics, Gülhane Health Sciences Faculty University of Health Sciences Ankara Turkey; ^2^ Department of Nutrition and Dietetics, Gülhane Health Sciences Institute University of Health Sciences Ankara Turkey; ^3^ Department of Nutrition and Dietetics Gülhane Health Sciences Faculty, University of Health Sciences Ankara Turkey

**Keywords:** anxiety, depression, food addiction, hedonic hunger, stress, ultra‐processed food consumption

## Abstract

Ultra‐processed foods (UPF) play a central role in modern diets but pose a serious threat to public health. The aim of this study was to investigate the relationship between UPF consumption and hedonic hunger, food addiction, and mood and to explain the impact of these factors on dietary habits. This cross‐sectional study included 3997 adults (2517 female, 1480 male), aged 18–65 years, recruited through snowball sampling in Ankara, Turkey. Data were collected via a web‐based survey that included demographic information, self‐reported anthropometric measurements, and validated scales: Screening Questionnaire of Highly Processed Food Consumption, The Power of Food Scale, Yale Food Addiction Scale, and Depression Anxiety Stress Scale‐21. The mean age of the participants was 31.7 ± 12.82 years. The Screening Questionnaire of Highly Processed Food Consumption was positively correlated with the Yale Food Addiction Scale score and the subdimension scores of the Depression Anxiety Stress Scale‐21 (*p* < 0.05). According to linear regression analysis, it was determined that gender, age, Yale Food Addiction Scale score, and the scores of depression, anxiety, and stress affected the Screening Questionnaire of Highly Processed Food Consumption score (*p* < 0.05). There was a difference between the groups with low and high levels of UPF consumption in terms of age, gender, marital and working status, Yale Food Addiction Scale score, and the subdimension scores of the Depression Anxiety Stress Scale‐21 (*p* < 0.05). In conclusion, considering the influence of food addiction and mood on UPF consumption, policies and interventions targeting the psychosocial processes of individuals should be developed and further research conducted in this area to limit the UPF consumption and promote healthy eating habits along with conscious eating behaviors.

## Graphical abstract

According to linear regression analysis, it was determined that gender, age, Yale Food Addiction Scale score, and the scores of the depression, anxiety, and stress affected the Screening Questionnaire of Highly Processed Food Consumption score (*p* < 0.05). There was a difference between the groups with low and high levels of UPF consumption in terms of age, gender, marital and working status, Yale Food Addiction Scale score, and the subdimension scores of the Depression Anxiety Stress Scale‐21 (*p* < 0.05).

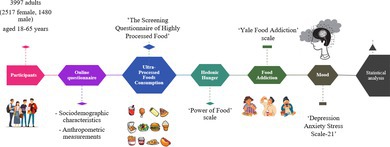

## 1 Introduction

Since prehistoric times, people have struggled with many environmental challenges to survive, and nutrition has become a fundamental element of survival in this process (Yılmaz 2023). While the primary purpose of nutrition in ancient times was only to fill the stomach, these habits have transformed over time under the influence of environmental, cultural, and technological factors and have formed the basis of today's understanding of nutrition (Pekcan et al. 2008). Food processing methods, which have become widespread, especially with the development of technology (Yılmaz 2023), have caused industrially processed foods to play a central role in modern nutritional habits (Bonaccio et al. 2021).

The degree of processing of foods and the purpose of this process have become an important determinant of population health by affecting the nutrient profile of foods, and therefore, the quality of diet. In this context, the NOVA classification system offers an internationally accepted method by grouping foods according to the nature and scope of industrial processing (Ülker and Çamli 2024). According to the NOVA classification, foods are divided into four main groups according to their degree of processing and intended use (Monteiro et al. 2018a). Ultra‐processed foods (UPF), one of these categories, are defined as foods whose natural structures have deteriorated, contain many additives, and have undergone intensive industrial processing (Günay 2024). Ingredients such as anti‐humectants, emulsifiers, and flavor enhancers are commonly used in the production of UPFs, enabling these products to achieve characteristics such as extended shelf life, low production cost, and intensified flavor (Monteiro et al. 2018a).

The advantages of UPFs such as practicality and attractiveness, combined with the acceleration of urbanization, increased participation of women in business life, and aggressive advertising campaigns of food companies, have caused the consumption of these foods to become widespread (Oruçoğlu et al. 2024). However, the high energy and saturated fat content of UPFs, low nutritional value (Monteiro et al. 2019), and addictive properties (Prescott et al. 2024) pose a serious threat to public health by causing health problems such as obesity, diabetes, and cancer (Oruçoğlu et al. 2024; Okyar et al. 2023). It is also stated that UPF consumption does not only cause physical health problems but also affect individuals' psychosocial processes, such as hedonic hunger, food addiction, and mood (Monteiro et al. 2019; Prescott et al. 2024; Okyar et al. 2023; Doğan 2024).

Food addiction is defined as an adaptation to certain foods that individuals are hypersensitive to and regularly consume and is included in the DSM‐V (Gearhardt et al. 2011a; Randolph 1956). This addiction is usually seen in foods rich in carbohydrates and fats (Cope and Gould 2017; Akten and Noyan 2019). A limited number of studies have shown that UPFs may be associated with food addiction and may cause addiction symptoms such as deprivation, excessive desire, and uncontrolled consumption in individuals due to their refined carbohydrate and fat content (Schulte et al. 2015; Wiss and LaFata 2024). In addition to the addictive effect of these foods, it has been seen that they may also be related to the concept of hedonic hunger by affecting the brain's reward system (Armutçu 2021). However, there is no study examining the effect of hedonic hunger on UPF consumption.

Hedonic hunger is characterized by increased reward sensitivity, pleasure, and food drive in the absence of physiological hunger (Mason et al. 2020). In the development of hedonic hunger, neural systems play an important role (Lee and Dixon 2017). In particular, brain regions such as the amygdala, hippocampus, and orbitofrontal cortex, where cannabinoid receptor and opioid signals are linked, play a critical function in the neural processes associated with hedonic hunger (Takeuchi 2021). UPFs can cause functional changes in the reward regions of the brain and increase endogenous opioid levels, thereby increasing food cravings (Armutçu 2021). The few studies in the literature that have examined the effect of hedonic hunger on UPF consumption suggest that there is a positive relationship between UPF consumption and hedonic hunger, and that individuals with hedonic hunger may be more likely to gravitate toward UPFs made palatable by high fat, sugar, and salt content (Doğan 2024; Gupta et al. 2020; Horwath et al. 2020).

Other changes in individuals' stress levels and mood can cause changes in appetite (Şarahman Kahraman and Akçil 2022). It is known that eating behavior, especially in individuals with high levels of stress, affects neuroendocrine mechanisms such as the hypothalamic–pituitary–adrenal axis, making the brain's reward center more sensitive and can create a positive feedback loop with the opioid stimulation provided by UPFs (Adam and Epel 2007; Wei et al. 2019). This shows that individuals with mood disorders such as depression, anxiety, and stress may generally prefer UPFs (Ülker and Çamli 2024).

In this context, examining the food addiction, hedonic hunger, and mood factors that may affect UPFs consumption will provide a deeper understanding of individuals' eating behaviors and contribute to obtaining comprehensive information about the psychosocial factors that shape these behaviors. Since the number of studies on psychosocial factors affecting UPFs consumption is limited in the literature, it is thought that this study will contribute to the literature. The main purpose of the study is to examine the effects of hedonic hunger, food addiction, and mood on UPF consumption.

## 2 Materials and Methods

**FIGURE 1:**
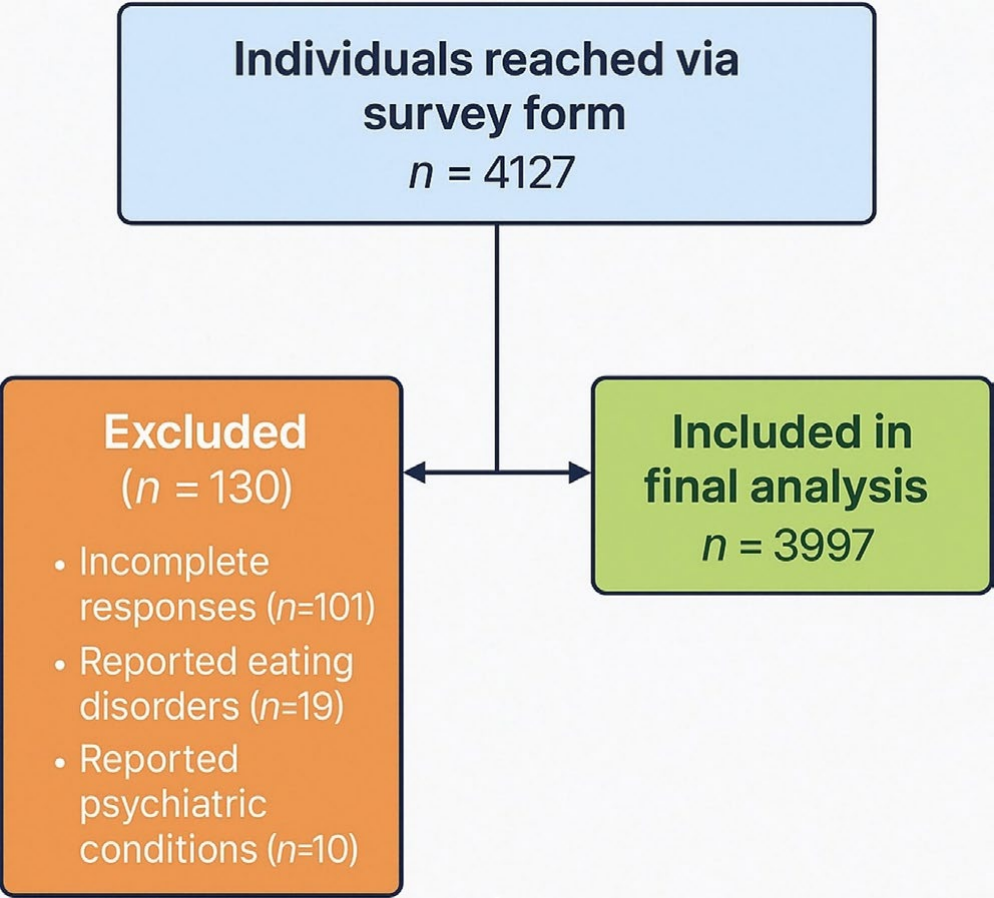
Study flowchart.

This cross‐sectional study was conducted among 3997 adults (2517 female, 1480 male) aged 18–65 years between September 2024 and January 2025. The research data were collected via a web‐based survey form (Google Forms) created by the researchers and taken from the Ankara province of Türkiye using the snowball sampling method. Snowball sampling, although a non‐probability method, has been reported as a practical approach in online health behavior studies to reach large and diverse samples in a cost‐effective manner (Naderifar et al. 2017). The survey data were collected via the social media tools Twitter, Facebook, WhatsApp, and Instagram. The inclusion criteria of the study were as follows: Individuals aged between 18 and 65, with internet access, who checked the “I agree to participate in this study voluntarily” tab at the beginning of the online survey and who completed the survey completely were included in the study. Participants with incomplete survey responses (*n* = 101), reports of eating disorders (*n* = 19), or history of psychiatric disorders (*n* = 10) were excluded from the study. A total of 4127 people were reached through the survey form; however, in line with the determined exclusion criteria, 130 participants were excluded from the study and the final analysis was carried out with a sample of 3997 participants (Figure 1).

Before starting the study, ethical approval with decision number 2024/541 from the University of Health Sciences Gulhane Scientific Research Ethics Committee was obtained. All study procedures were performed in compliance with the principles of the Declaration of Helsinki. The demographic characteristics (gender, age, education level, marital status, income status, and working status) and anthropometric measurements (body weight and height) of the individuals were questioned. Previous studies have validated the applicability of all scales measuring UPF consumption, hedonic hunger, food addiction, and mood within online research settings (Erdoğan Gövez et al. 2024; Varnado et al. 2024; Wittayapun et al. 2023; Howard et al. 2021).

### 2.1 Anthropometric Measurements

Anthropometric measurements, including body weight and height, were obtained through self‐report provided by the participants. Detailed written instructions were added to the anthropometric measurements section to minimize measurement error and ensure consistency. Participants were instructed to weigh themselves using a calibrated scale, after a fast of at least 4 h, without shoes and in light clothing. For the measurement of height, it was suggested that they stand upright with their backs against the wall without shoes and measure using a flat object (such as a book) aligned with the top of their head and with the help of another person. A visual was added on how to provide the Frankfurt plane. In order to standardize the process, explanatory pictures of all measurements were included in the questionnaire form and step‐by‐step guidance was provided. Body mass index (BMI) was calculated by dividing body weight (kg) by the square of height (m^2^). A BMI of < 18.50 kg/m^2^ was classified as underweight, 18.50–24.99 kg/m^2^ as individuals with normal weight, 25.00–29.99 kg/m^2^ as individuals with overweight, and 30.00 kg/m^2^ or higher as individuals with obesity (Madden and Smith 2016).

### 2.2 Ultra‐Processed Food Consumption

The Screening Questionnaire of Highly Processed Food Consumption was used to assess food consumption. The Turkish validity and reliability study of the scale was conducted by Erdoğan Gövez et al. (2024). The Cronbach's alpha coefficient of the scale is 0.65. The scale consists of 11 food items. If the participant answers “yes” to consumption, each food item is given 1 point, while if the participant answers “no,” each food item is given 0 points. The total score varies between 0 and 11. Scores of 6 and above on the scale are considered as high consumption. As the score increases, UPF consumption also increases.

### 2.3 Hedonic Hunger

The Power of Food Scale was used to assess hedonic hunger status. The reliability and validity of the Turkish version of the scale were established by Ulker et al. (2021). The Cronbach's alpha coefficient of the scale is 0.922. This 5‐point Likert‐type scale consists of 13 items. The total score is obtained by summing the item scores and dividing them by the number of items. An increase in the score indicates a greater tendency to hedonic hunger.

### 2.4 Food Addiction

The food addiction status of the individuals was evaluated with the Yale Food Addiction Scale. The Yale Food Addiction Scale was developed by Gearhardt et al. (2009). The Turkish validity and reliability study of the scale was conducted by Sevinçer et al. (2014). The Cronbach alpha coefficient value of the scale is 0.93. The scale consists of 27 items and is used to measure addiction‐like eating behaviors toward certain food types over the last 12 months. The scale was created by adapting the substance addiction criteria in the DSM‐IV to food addiction to determine the addiction to certain foods. The scale consists of eight symptoms. The scale score is calculated on the total score of the questions belonging to each symptom. Individuals with a symptom score ≥ 3 are diagnosed with food addiction, provided they score 1 point from one of the 15th and 16th questions of the scale.

### 2.5 Mood

The emotional states of the individuals were evaluated with the Depression Anxiety Stress Scale‐21. This scale was developed by Lovibond and Lovibond. The Turkish validity and reliability study of the scale were conducted by Yılmaz et al. (2017). The scale consists of three subdimensions: depression, anxiety, and stress.

The Cronbach alpha coefficient of the scale was found to be 0.94, 0.87, and 0.91 for the subdimensions, respectively. Each dimension contains seven items. The scale is a 4‐point Likert type, and individuals are asked to give a score between 1 and 4 for each item of the scale questions. As the score received from the subscales increases, the severity of the symptoms increases.

### 2.6 Statistical Analysis

The G*Power software (Version 3.1.9.6) was used to analyze the sample's size. The effect size of the study was aimed to be a poor or medium correlation between UPF consumption and food addiction. Based on an effect size of |*ρ*| = 0.20, correlation: point biserial model, according to the with two tail, alpha of 5%, power (1 − *β*) = 0.95, the sample size was calculated 726. The post doc analysis was performed after the study; based on the analysis (*r* = 0.225), the statistical power (1 − *β*) was found to be 98% for the statistical significance of a two‐sided alpha of 5%. The G*Power software is widely used in health research for a priori and post hoc power analysis to determine adequate sample sizes for detecting hypothesized effects (Faul et al. 2007).

All analyses were conducted using the Statistical Package for the Social Sciences software (version 22.0). Descriptive statistics, including mean, standard deviation, frequency, and percentage, were used to evaluate the data. Data distribution was assessed using histograms, the coefficient of variation, skewness, kurtosis, and Kolmogorov–Smirnov tests. Relationships between variables were determined using the Pearson correlation coefficient. Differences in mean values between groups were evaluated with the independent *t*‐test. Chi‐squared analysis was used to compare qualitative data between groups. Linear regression analysis was performed to predict UPF consumption. Results were interpreted at a 95% confidence interval with a significance level of *p* < 0.05.

## 3 Results

The general characteristics of the participants are shown in Table 1. Most of the participants were female (63.0%). The mean age was 31.7 ± 12.82 years, and the mean BMI was 24.5 ± 4.53 kg/m^2^. According to the BMI classification, 5.6% of the participants was classified as individuals with underweight, 55.2% as individuals with normal weight, 27.5% as individuals with overweight, and 11.7% as individuals with obesity. Most of the participants (52.9%) were unemployed. The mean score obtained from the Screening Questionnaire of Highly Processed Food Consumption was 5.1 ± 2.73. The mean score of the Power of Food Scale was 2.8 ± 1.03, and the mean score of the Yale Food Addiction Scale was 2.9 ± 1.65. The mean scores for depression, anxiety, and stress on the Depression Anxiety Stress Scale‐21 were 5.6 ± 4.76, 5.0 ± 4.38, and 5.9 ± 4.47, respectively. While 44.8% of the participants had a high UPF consumption, 55.2% had a low UPF consumption. While 86.7% of the participants were found to have food addiction, 13.3% were found not to have food addiction.

**TABLE 1:** General characteristics of individuals.

Variables	X¯ ± SD
Age (years)	31.7 ± 12.82
BMI (kg/m^2^)	24.5 ± 4.53
Number of main meals	2.4 ± 0.53
Number of snacks	1.5 ± 1.00
Screening Questionnaire of Highly Processed Food Consumption score	5.1 ± 2.73
Power of Food Scale score	2.8 ± 1.03
Yale Food Addiction Scale score	2.9 ± 1.65
Depression Anxiety Stress Scale‐21 score	
Depression score	5.6 ± 4.76
Anxiety score	5.0 ± 4.38
Stress score	5.9 ± 4.47
	Number (%)
Gender	
Female	2517 (63.0)
Male	1480 (37.0)
Education level	
Primary school	158 (4.0)
Middle school	199 (5.0)
High school	900 (22.5)
University	2541 (63.6)
Master's degree/Doctorate	199 (5.0)
Marital status	
Single	2391 (59.8)
Married	1606 (40.2)
Income status	
Income less than expenses	1060 (26.5)
Income equal to expenses	1798 (45.0)
Income more than expenses	1139 (28.5)
BMI classification	
Underweight	224 (5.6)
Normal	2207 (55.2)
Overweight	1098 (27.5)
Obese	468 (11.7)
Working status	
Yes	1881 (47.1)
No	2116 (52.9)
Ultra‐processed food consumption	
High consumption	1789 (44.8)
Low consumption	2208 (55.2)
Food addiction	
Yes	3465 (86.7)
No	532 (13.3)

The relationship between the Screening Questionnaire of Highly Processed Food Consumption, Power of Food Scale, Yale Food Addiction Scale, and Depression Anxiety Stress Scale‐21 is shown in Table 2. A positive significant relationship was found between the Screening Questionnaire of Highly Processed Food Consumption score and the Yale Food Addiction Scale score (*r* = 0.225, *p* < 0.001). Similarly, the Screening Questionnaire of Highly Processed Food Consumption score showed positive significant relationships with the subdimension scores of the Depression Anxiety Stress Scale‐21 (*r* = 0.218; *p* < 0.00, *r* = 0.223; *p* < 0.00, *r* = 0.223; *p* < 0.001, respectively).

**TABLE 2:** The relationship between Screening Questionnaire of Highly Processed Food Consumption, Power of Food Scale, Yale Food Addiction Scale, and Depression Anxiety Stress Scale‐21.

	Screening Questionnaire of Highly Processed Food Consumption score
Power of Food Scale score	*r* = 0.019 *p* = 0.240
Yale Food Addiction Scale score	*r* = 0.225 *p* < 0.001*
Depression Anxiety Stress Scale‐21	
Depression score	*r* = 0.218 *p* < 0.001*
Anxiety score	*r* = 0.223 *p* < 0.001*
Stress score	*r* = 0.223 *p* < 0.001*

* *p* < 0.05.

Evaluation of individuals' characteristics according to UPF consumption is shown in Table 3. The mean age of individuals with high UPF consumption was lower (28.8 ± 11.75 years), whereas the mean age of participants with low UPF consumption was higher (34.1 ± 13.17 years). A statistically significant difference was found between low and high consumption in terms of gender distribution (*p* < 0.001). 59.1% of female participants and 40.9% of male participants had high UPF consumption. A statistically significant difference was found between low and high consumption in terms of marital status (*p* < 0.001). High UPF consumption was found to be higher in single participants (68.6%) than married participants (31.4%). When working status was examined, it was seen that unemployed individuals (55.4%) had higher UPF consumption (*p* = 0.002). There was no significant difference between the groups in terms of the number of main meals and snacks, BMI classification, and the Power of Food Scale score. A significant difference was observed in the Yale Food Addiction Scale score between the groups (*p* < 0.001). The mean score of the Yale Food Addiction Scale was higher in participants with high UPF consumption (3.3 ± 1.79). In addition, the scores of depression, anxiety, and stress were found to be significantly higher in participants with high UPF consumption (*p* < 0.001).

**TABLE 3:** Evaluation of individuals' characteristics according to ultra‐processed foods consumption.

Variables	Highly processed food consumption		*p*
	High consumption (*n* = 1789)	Low consumption (*n* = 2208)	
Age (years)	28.8 ± 11.75	34.1 ± 13.17	< 0.001*
Gender			
Female	1057 (59.1)	1460 (66.1)	< 0.001*
Male	732 (40.9)	748 (33.9)	
Education level			
Primary school	50 (2.8)	108 (4.9)	0.056
Middle school	78 (4.4)	121 (5.5)	
High school	398 (22.2)	502 (22.7)	
University	1197 (66.9)	1344 (60.9)	
Master's degree/Doctorate	66 (3.7)	133 (6.0)	
Marital status			
Single	1228 (68.6)	1163 (52.7)	< 0.001*
Married	561 (31.4)	1045 (47.3)	
Income status			
Income less than expenses	506 (28.3)	554 (25.1)	0.067
Income equal to expenses	779 (43.5)	1019 (46.2)	
Income more than expenses	504 (28.2)	635 (28.8)	
Working status			
Yes	797 (44.6)	1084 (49.1)	0.002*
No	992 (55.4)	1124 (50.9)	
Number of main meals	2.5 ± 0.53	2.5 ± 0.53	0.996
Number of snacks	1.6 ± 1.02	1.4 ± 0.97	0.146
BMI (kg/m^2^)	24.5 ± 4.72	24.4 ± 4.37	0.699
BMI classification			
Underweight	110 (6.1)	114 (5.2)	0.250
Normal	970 (54.2)	1237 (56.0)	
Overweight	470 (26.3)	628 (28.4)	
Obese	239 (13.4)	229 (10.4)	
Power of Food Scale score	2.8 ± 1.05	2.8 ± 1.02	0.427
Yale Food Addiction Scale score	3.3 ± 1.79	2.7 ± 1.48	< 0.001*
Depression Anxiety Stress Scale‐21 score			
Depression score	6.5 ± 4.96	4.9 ± 4.47	< 0.001*
Anxiety score	5.8 ± 4.53	4.3 ± 4.12	< 0.001*
Stress score	6.8 ± 4.62	5.2 ± 4.21	< 0.001*
Food addiction			
Yes	322 (18.0)	210 (9.5)	< 0.001*
No	1467 (82.0)	1998 (90.5)	

* *p* < 0.05.

Linear regression analysis was performed to evaluate the factors that could affect the Screening Questionnaire of Highly Processed Food Consumption score, and the model was found to be significant (*R* ^2^ = 0.353; *p* < 0.001). It was determined that gender, age, Yale Food Addiction Scale score, and the scores of the depression, anxiety, and stress affected the model (*p* < 0.05) (Table 4).

**TABLE 4:** Linear regression model for highly processed food consumption prediction.

Model	Screening Questionnaire of Highly Processed Food Consumption score		
	Beta	*t*	*p*
Gender	−0.199	−13.227	< 0.001*
Age (years)	−0.101	−6.758	< 0.001*
Yale Food Addiction Scale score	0.165	10.447	< 0.001*
Depression score	0.139	8.769	< 0.001*
Anxiety score	0.151	9.336	< 0.001*
Stress score	0.150	9.482	< 0.001*
		*R* ^2^ = 0.353; *p* < 0.001*	

*Note:* Variable values: Gender (Male = 1, Female = 0).

* *p* < 0.05.

## 4 Discussion

In this study, hedonic hunger, food addiction, and mood factors affecting UPF consumption were examined. The main findings showed that individuals with higher UPF consumption had significantly higher food addiction and negative mood scores. Furthermore, UPF intake was more common among young adults, women, single individuals, and unemployed individuals, and food addiction and mood symptoms were more intense in individuals with higher UPF consumption. Regression models showed that UPF consumption was inversely associated with age and gender, while it was positively associated with food addiction and mood symptoms.

Global consumption of UPFs and the percentage of energy coming from UPF consumption are increasing significantly (Zhang and Giovannucci 2023; Martini et al. 2021). Many factors, such as income growth, urbanization, changing workforce structures, and demographic characteristics in developed and developing countries, play a role in this increase in UPF consumption (Baker et al. 2020). However, UPFs, which stand out in their practicality, high palatability, and low cost, play an important role in shaping the psychological state of individuals and causing changes in their eating behavior (Monteiro et al. 2018a; Prescott et al. 2024; Wiss and LaFata 2024; Sen et al. 2024; Melekoğlu 2021).

The findings of the study that individuals with high UPF consumption are mostly women and that UPFs consumption is reported more in the Screening Questionnaire of Highly Processed Food Consumption scores than in men underline the gender differences in UPF consumption. In parallel with the results of this study, a study examining UPF consumption and socio‐demographic factors found that UPF consumption by women was statistically significant compared to men (Khandpur et al. 2020). A different study conducted in 2024 found that the subjects with high UPF consumption were women (Hosseinpour‐Niazi et al. 2024). Gender differences, along with the increasing participation of women in the workforce, have contributed to the growing appeal and suitability of ready‐to‐eat and ready‐to‐heat food options (Monteiro et al. 2013). In addition, nutrition is now seen by many people as a method of coping with a negative situation or emotion, or as a rewarding method (Bilici et al. 2020). Accordingly, the tendency toward foods with high sugar content increases in stressful situations (Melekoğlu 2021), and this situation is also associated with the symptoms of craving for sweet, tasty foods in women (Lustig 2020). The absence of preparation and cooking stages in UPFs has also been identified as a contributing factor to this outcome (Melekoğlu 2021). It has also been thought that these gender differences may be because men are more likely to describe unhealthy foods as healthy than women (Nelson and Fleming 2019).

When the relationship between marital status and UPFs was examined, it was seen that the relationship between single status and high UPF consumption was significant. Similar results were observed in a study based on the Turkey Nutrition and Health Survey data, and it was found that marital status affected UPF consumption, with single individuals consuming more UPFs than married individuals (Aciduman Subaşiay 2022). Similar results were observed in another study conducted in Spain, where single individuals consumed more UPFs (Gómez‐Donoso et al. 2020). This relationship has been attributed to the tendency of these foods to meet nutritional needs quickly (Aciduman Subaşiay 2022). It is thought that this may also be due to the different lifestyles and eating habits of single individuals.

In studies examining the relationship between age and UPF consumption, data from different countries are noteworthy. In Portugal, the National Survey on Food, Nutrition and Physical Activity, conducted in 2015–2016, examined the sociodemographic characteristics of 5005 participants. In this study, it was determined that younger age groups, especially adolescents, consumed more UPFs. Data from the internet‐based NutriNet‐Santé study, launched in France from May 2009 to June 2014, also showed similar results. This study assessed the socio‐demographic characteristics of 74,470 participants, together with 24‐h dietary records. As a result of the study, it was determined that younger age groups consumed more UPFs (Magalhães et al. 2021). In a systematic review evaluating studies conducted up to 2021 and including a total of 99 studies, the mean age of participants was 38.9 years and 58.8% were female. In this review, UPF consumption generally decreased with age and was most common in children and adolescents and least common in the elderly (Marino et al. 2021). Our results are consistent with these findings, showing that the mean age was lower in high UPF consumption than low UPF consumption. According to our linear regression model, the negative association of age with UPF consumption suggests generational differences in dietary habits (Casini et al. 2015). The negative association of UPF intake with age may be influenced by differences in age‐specific food preferences (e.g., younger children consumed more fruit juice/beverages and cakes, biscuits and other pastries than other age groups, while adolescents and younger adults consumed more fast food, frozen meals, and soft drinks). These preferences may be explained by the increased dietary requirements of younger generations such as Generation Z, peer social pressure, limited cooking skills, and the affordability of UPFs (Monteiro et al. 2018b; Luszczynska et al. 2013; Janssen et al. 2018). In addition, the presence of UIG advertising in school meals and commercials may also explain this association (Aciduman Subaşiay 2022). In this context, practices such as intergenerational nutrition interventions, school feeding programs, and advertising media may also be considered by policymakers to increase the effectiveness of public health initiatives (Aciduman Subaşiay 2022; Casini et al. 2015).

Another important finding of the study is the positive relationship between UPF consumption and food addiction. Participants with high UPF consumption were also found to have a high level of food addiction. This relationship, measured with the Yale Food Addiction Scale, supports studies in the literature suggesting that the high refined carbohydrate and fat content of UPFs may lead to changes in the brain's reward‐punishment system, leading to the emergence of addictive eating behaviors (Schulte et al. 2015; Gordon et al. 2018; Canhada et al. 2020). The association of UPFs with food addiction is also associated with the increasing prevalence of noncommunicable diseases (Askari et al. 2020; Capra et al. 2022) In addition, the high addictive potential of UPFs has been attributed to the individual or combined effects of sugar (Lustig 2020) and fat (Schulte et al. 2015). Given the association of UPFs with food addiction, it is of great importance to promote healthy eating habits, identify a potentially addictive profile of certain foods, and provide public health education (Gearhardt et al. 2013; Gearhardt and Brownell 2013; Gearhardt et al. 2011b).

Furthermore, the significant association between UPF consumption and mood factors such as depression, anxiety, and stress highlights the complex interaction between diet and emotional health. In the study, depression, anxiety, and stress scores were higher in individuals with higher UPF consumption. This significant association highlights the bidirectional relationship between emotional well‐being and diet (Adjibade et al. 2019). It also suggests that UPFs may be used as a coping mechanism for negative emotional states (Hecht et al. 2022). However, it should be noted that they may exacerbate mental and physical health problems in the long term (Sen et al. 2024). Studies in the literature have shown similar results to the present study and have found an association with increased stress levels; increased consumption of sugary foods, fast food, and snacks; and decreased consumption of meat, fish, and vegetables, and fresh fruit at main meals (Kandiah et al. 2006; Oliver and Wardle 1999). Two prospective cohort studies, including 183,439 individuals, found an increased risk of depression with higher UPF consumption (Adjibade et al. 2019). Another study from 2024, which assessed UPFs and emotional states, observed significant associations between anxiety and UPF consumption (Ülker and Çamli 2024). As a result, individuals with mood swings were reported to be more likely to consume UPFs, which are often made palatable by their high fat, sugar, and salt content (Lowe and Butryn 2007; Gearhardt and Schulte 2021). Taken together, UPF consumption is positively associated with the risk of developing depressive symptoms, suggesting that addressing this non‐nutritive aspect of the diet may be necessary to improve mental health (Adjibade et al. 2019).

In general, high UPF consumption is associated with increased food addiction and mood. In this study, unlike other studies, no relationship was found between UPF consumption and the hedonic hunger of the participants. It stated in the literature that the hedonic properties of UPFs, which are associated with high fat, sugar and salt content, may cause consumers to continue eating these foods even when they are full because the “pleasure of eating” will dominate the feeling of satiety (Fardet and Rock 2019). Similarly, most studies have observed significant relationships between UPFs and hedonic hunger. However, possible reasons for these inconsistencies in our study may be the observational nature of the study and its limited duration, the physiological complexity, and the interactions of various factors affecting food intake and metabolism (Doğan 2024). In addition, there is no information about the participants' level of awareness or knowledge about nutrition in this study. Therefore, participants with low UPFs consumption may have preferred a diet rich in UPFs.

The main strength of this study is to jointly evaluate the impact of food addiction, hedonic hunger, and mood on UPFs consumption. Furthermore, the large sample size strengthens the statistically significant results of the study. The study has some limitations. One of the most important limitations of this study is the use of a non‐probabilistic snowball sampling method, which may have introduced selection bias and affected the generalizability of the findings. Although it enabled access to a large and diverse sample via online platforms, it does not guarantee that every individual in the target population had an equal chance of being included, which limits the representativeness of the results. Second, the cross‐sectional nature of the study may prevent causal inferences between the variables. Therefore, longitudinal studies are needed to determine the direction of the observed relationships. Third, participant‐reported data may lead to reporting bias and produce unreliable results. Fourth, this study conducted in Türkiye may not fully reflect socioeconomic or cultural differences in UPFs consumption patterns. Finally, although the primary aim of this study was to identify factors affecting UPFs consumption, future studies may consider constructing separate regression models in which UPF intake is treated as an independent variable and psychological scales (such as food addiction, depression, anxiety, and stress) as outcomes. This approach would allow for a more comprehensive evaluation of bidirectional associations between diet and mental health.

## 5 Conclusion

Tthe current study highlights the impact of food addiction and mood on UPF consumption. In addition, age, gender, marital, and working status were found to be factors associated with UPF consumption. This study provided a better understanding of the effects of eating behaviors and moods on UPF consumption. The results suggest that UPF consumption is used as a negative coping mechanism. UPF consumption should be addressed not only in terms of individual health but also in terms of public health policies and interventions. In this context, it is important to promote healthy eating habits; limit access to high‐calorie, low‐nutrient foods; and increase awareness programs to support conscious eating behavior.

## Author Contributions

**Özge Mengi Çelik:** conceptualization, data curation, formal analysis, supervision, validation, writing – original draft, writing – review and editing; **Ümmügülsüm Güler:** conceptualization, data curation, writing – original draft; **Emine Merve Ekici:** conceptualization, data curation, writing – original draft.

## Conflicts of Interest

The authors declare no conflicts of interest.

## Data Availability

The data that support the findings of this study are available from the corresponding author upon reasonable request.
